# A Short History of the B-Cell-Associated Surface Molecule CD40

**DOI:** 10.3389/fimmu.2014.00472

**Published:** 2014-09-29

**Authors:** Edward A. Clark

**Affiliations:** ^1^Department of Immunology, University of Washington, Seattle, WA, USA

**Keywords:** B cell, CD40, monoclonal antibody, activation, therapy

## Abstract

This perspective traces developments using monoclonal antibody technology that led to the discovery of CD40, a receptor that on B cells mediates “T cell help” and on dendritic cells helps to program CD8 T cell responses. I discuss some things that we got right during the path of discovery and some things we missed. Immunotherapies that block or stimulate the CD40 pathway hold great promise for treatment of autoimmune diseases and cancers.

During the late 1970s, a number of immunology labs started using the new hybridoma technology to establish monoclonal antibodies (mAbs) and define receptors expressed at different stages of hematopoietic cell differentiation. A central premise of this field was based on the seminal studies of Ted Boyse, Lloyd Old, and their colleagues (1, 2). They used antisera to define what they termed “differentiation antigens” (Ags), which unlike histocompatibility (H) antigens, were a “source of antigenic variation within the species that between different cell types in a single individual” and thus were “recognizable only with antiserum prepared in a foreign species” (1). The groundwork was laid for trying to make mouse mAbs to differentiation Ags that could be used to define stages of lymphocyte differentiation and distinct functional cell subsets, an approach I used to make an mAb to what is now called CD40 (3).

As a postdoc in London with Av Mitchison, I had made mAbs, including some to the mouse T-cell marker, Thy-1. Upon arriving in Seattle, I decided to make mAbs to human B-cell differentiation Ags. To identify markers conserved in evolution, we immunized mice with a baboon B-cell line and screened for mAbs that reacted with human B-cell lines but not other lymphoid or non-lymphoid lines. One mAb that reacted just with B lymphoblasts but not T-cell blasts called BB1 (4), Peter Linsley later used BB1 to define CD80 (B7/BB1) as a ligand for CD28 (5).

We also immunized mice with human B cells and selected mAbs that bound to human B cells and not T cells. In 1982, we submitted a set of our mAbs to an international workshop established to classify mAbs binding to human leukocyte differentiation Ags. A major impetus for this first workshop came from scientists who had organized workshops to define and standardize HLA antigens and HLA typing. As had been the case for HLA nomenclature, a neutral nomenclature was needed for human differentiation antigens, particular since markers like CD4 were called different names-T4, OKT4, Leu3, etc., depending on the company that was hawking the mAb for sale. The HLA serologists proposed a serological approach to define so-called “clusters of differentiation” (CD) to identify and name human differentiation markers. I had started out working in a HLA serology lab with Paul Terasaki at UCLA. But I was influenced by a group of scientists with strong biochemical backgrounds, particularly by Jeff Ledbetter and also later by Tucker LeBien and Bob Knowles, who felt that it was very important to use a combination of biochemistry and serology to define the new CD molecules. So, we decided to use the molecular weight of any new molecule when naming it. One B-cell-restricted 35-kDa molecule we called Bp35 (later designated CD20), while another 76-kDa molecule expressed on activated lymphocytes we called p76 (later designated CD54/ICAM1). Using this approach, by late 1982, we were able to conclude that “there are now over 12 different human B-cell-associated antigens distinct from immunoglobulin” (6). What could all their functions be?

At the time of our discovery of CD40 (3), immunologists were just beginning to understand how B cells are activated to divide and differentiate. One widely held model was that, after an initial activation signal, e.g., by a low dose of Ag or other means of B-cell receptor (BCR) crosslinking, B cells were activated and then expressed receptors for growth and differentiation factors such as “B-cell growth factor” (BCGF) and “B-cell differentiation factor” (BCDF) derived from T cells, the precursors of IL-4, IL-6, and IL-21, so that in the presence of these factors, B cells would divide and differentiate (7, 8). A receptor for the “T-cell growth factor,” IL-2, had been described (9), but not a BCGF receptor B-cell equivalent.

Boyse and his colleagues (10) and Subbarao and Mosier (11) had used mAbs to define a B-cell-restricted surface molecule in mice, Lyb-2 (later designated CD72), which when ligated with an mAb-induced B cells to proliferate but blocked their differentiation. Following this lead, we decided to test if any of our mAbs could activate human B cells. Geraldine Shu, a research scientist in the lab found that one of our Bp35 (CD20) mAb, 1F5, induced B cells to proliferate as measured by ^3^H-thymidine uptake, while another Bp35 mAb did not (12). We proposed that Bp35 might function as a receptor for a “second signal” distinct from the BCR, perhaps for a macrophage factor (13). In an important follow-up study, Golay and Beverley found that 1F5 anti-Bp35 (CD20) could induce resting G0 B cells to enter the G1 phase of the cell cycle (14). Similar to our results (12), they found that a BCGF could synergize with 1F5, but alone the BCGF could not activate resting B cells. Several groups had shown that two distinct kinds of signals were required for fibroblastic cells in G0 to transit through the cell cycle – a “competence” signal such as PDGF to induce cells in G0 to enter G1 and a “progression” signal such as EGF to drive cells through S phase (15, 16). Thus, we postulated that after B cells are stimulated via a Bp35 “competence” signal, they become responsive to a BCGF “progression” signal.

During 1985, we defined another set of anti-B-cell mAbs after immunizing mice with human tonsillar B cells. We enriched for B cells by depleting rosetting T cells out with sheep red cells, since magnetic beads, let alone magnetic bead kits, were not yet available. We screened the mAbs for their ability to induce B cells to proliferate and found that one of them, G28-5, dramatically increased B-cell proliferation, but only when anti-mu sera or 1F5 anti-Bp35 (CD20) was present. Importantly, G28-5 behaved like a “progression” signal in that it could augment B-cell proliferation even when added 48 h after the initial activation signal. Biochemical analyses using radioactive ^125^I-labeled tonsillar cells showed that G28-5 detected a 50-kDa protein on B cells, which we called Bp50. We immediately thought that we most likely had discovered a receptor for either “a soluble growth factor or for a signal mediated through cell-cell contact” that induces a “progression” signal in B cells and that the factor might be made by T cells (3).

The G28-5 mAb ended up have a number of uses: Ivan Stamenkovic in Brian Seed’s lab used G28-5 and expression cloning to isolate a cDNA encoding human CD40 (17). CD40 turned out to be related to the nerve growth factor receptor (NGFR), and after other similar receptors were identified, the receptor group was called the TNF receptor superfamily. It could just as easily been called the NGFR family after the first member cloned. We used the human CD40 cDNA sequence to isolate the mouse CD40 cDNA (18), and transfectants expressing CD40 were then used to characterize a number of useful anti-mouse CD40 mAbs.

CD40 mAbs were also used to characterize a signaling pathway that is both distinct from the BCR pathway and acts synergistically with BCR signaling; these studies help to stimulate investigation and understanding of TRAF protein-regulated signaling pathways (19). CD40 ligation was shown to activate CD18/CD11a-mediated adhesion to increase IL-6 expression, and together with IL-4 to induce isotype class switching to IgE, all before the ligand for CD40 was discovered (20–22). Liu et al. (23) made the important finding that G28-5 prevented B cells from dying, laying the groundwork for a role for CD40 in germinal center (GC) formation and studies of how lymphocytes are protected from cell death. Thus, many features and functions of the CD40 signaling pathway in B cells were made prior to the discovery of CD40L (24–26) and linking CD40 to “T-cell help.”

## What Did We Get Right?

The hypothesis that B cells, like fibroblasts (15, 16), need two signals – a “competence” signal and a “progression” signal – to proliferate seems to hold true. In general, lymphocytes need at least two signals to divide and differentiate. The focus on the function of a receptor expressed on B cells, rather than using new mAbs to CD markers to subdivide lymphocytes into ever increasing species and subspecies – the cellular immunologist’s perpetual distraction – clearly led to new insights. Focusing on a receptor’s function is all the more relevant in the current world of immunological research, where each month brings new data underscoring the plasticity of lymphocytes, their ability to adapt and change in a range of environments (27, 28).

Looking back, I am amazed at how much others and we were able to discover simply by using a mAb to a particular receptor expressed on B cells. The CD40 mAb, not the cDNA, paved the way to understanding what CD40 is and does. In our first paper on CD40 (3), we concluded that “this work in turn may help in devising strategies *in vivo* for the control of human diseases such as B-cell malignancies, immune-deficiencies, and certain autoimmune diseases.” Almost 30 years later, CD40 holds tremendous potential as a target for immunotherapeutics and vaccines.

Another important thing we got right was to send our CD40 mAb and other mAbs to whomever wanted them, usually as milligrams of purified protein without any strings attached, unless very large quantities were requested. We began this practice in the early 1980s and the number of requests steadily rose until by 1991 we were shipping out over 100 shipments of mAbs per year. We sent G28-5 to more than 100 labs, once to 11 labs on one day in 1993. This was at a time when there were few companies from which one could buy mAbs and none were selling anti-CD40; we felt it was our responsibility to get all the mAbs that we could out to those who could use them. I had learned the practice of open giving of reagents and ideas in science from Av Mitchison and Martin Raff in London. However, eventually we were tired of spending so much time and effort distributing mAbs. The companies that have taken over this task have done scientists a service, but of course instead of receiving milligrams of free mAb, we buy micrograms of conjugated mAbs at $350 or more a pop. I feel better when I am given something by a neighbor grown in her garden, instead of buying it at the store. The practice of science simply feels more personal when labs exchange gifts with each other without strings attached.

## What Did We Miss or Not Get Right?

While in Osaka in 1987 on sabbatical in Tadamitsu Kishimoto’s lab, two students, Seiji Inui and Tsuneyasu Kaisho, and I used the new CD40 cDNA to express wildtype (WT) human CD40 and CD40 mutants in a mouse cell line M12. We found that residue T234 in the CD40 tail is essential for CD40 signaling regulating cell survival (29). M12 cells expressing WT CD40 (M12-CD40) responded to anti-CD40 with growth inhibition while cells expressing CD40 without its cytoplasmic tail (M12-tailless) did not. I decided that this pair would be ideal for identifying the ligand for CD40 (CD40L). But none of the various BCGF and BCDF that we tested inhibited the growth of M12–CD40 but not of the M12-tailless cells. Cosman and his colleagues at Immunex in Seattle had set up a system where groups of cDNAs from a cDNA library were transiently transfected in Cos cells and supernatants screened for activity. We began collaborating with Immunex and tested a large number of supernatants from transfected cells for their ability to inhibit M12–CD40 cells but not M12–tailless cells. We were very excited when within a few months we identified a candidate supernatant that had the properties we were looking for. However, when the cDNA encoding the protein was sequenced, it turned out to encode for mouse IL-4. This was quite surprising not only because a mouse cDNA was picked up in a screen from a human cDNA library. How could it be that mouse IL-4 (and not human IL-4 we subsequently discovered) of all factors signaled cells expressing WT CD40 but not cells missing the CD40 tail? For more than a year, we tested everything we could get our hands on using the M12 screening assay including supernatants from stromal cells for possible “CD40L activity,” all to no avail. I became disheartened, and we stopped working on the project. The Immunex team to their credit persevered and using another approach was able to discover CD40L (24). Although I had helped to initiate the search for CD40L, by focusing on the screen on M12 cell lines, I missed the chance to be actively involved in discovering it. The discoveries of CD40L, the CD40L defects in patients with hyper-IgM syndrome and subsequent studies with CD40- and CD40L-deficient mice established the key role of CD40 in T-cell-dependent B-cell responses (24–26, 30).

In our early publications, we focused on the role of CD40 on B cells, even though others and we early on had found that CD40 is expressed on epithelial cells and carcinomas (31). Ling et al. (32) in the third CD workshop in 1986 unequivocally showed that CD40 was expressed on interdigitating cells in T-cell zones, and Hart reported in 1988 that CD40 is expressed on human tonsillar dendritic cells (DCs) (33). In spite of knowing that CD40 was expressed on DCs, we did not test whether G28-5 could stimulate DCs for many years. We were simply too B-cell-centric! Only when Rainheim and Kipps (34) reported that CD40 ligation upregulates the expression of CD80 on B cells did we finally get around to testing if that was the case for DCs (35). By then, a number of groups were investigating the function of CD40 on DCs (36, 37); important discoveries followed, not the least of which was the discovery that CD40 signaling plays a vital role in programing CD8 T-cell responses (38). So, we missed a chance to define some key CD40 functions in DCs.

Nevertheless, we began to appreciate the active communication between T and B cells more fully and the links between the CD40L–CD40 signaling and CD80–CD28 signaling (39). CD4 T cells then and even today are sometimes referred to as “conductors”, as the cells that “orchestrate” immune responses, a view bolstered not only by paternalistic thinking immunologists but also by the fact that the dreaded HIV-1 virus targets CD4 T cells. I thought of the program orchestrated by T cells and B cells as being based more on a constructive and mutual conversation (39) between the sometimes underappreciated “female” B cells and respectful “male” T cells, rather a program conducted by dominant CD4 T cells. Some days, it felt like the field of immunology was full of chauvinists who thought that “male” CD4 T cells were running the show and thus the only cells worth studying. Even today they are still 10 times or more NIH grants funded on CD8 T-cell memory than on B-cell memory, even though both cytotoxic T cells and antibodies play key roles in sustained protective immunity.

An effective outcome requires quality communication between the parties involved. We proposed that T and B cells communicate via “reciprocal dialogs” between equally important cells and involving the CD40L–CD40 and CD80–CD28 “phrases” or pathways that reinforce each other (39). I drew the diagram made to illustrate this point purposely as a yin-yang symbol with Ag-MHC class II peptide/TCR in the middle. Today, B cells are better appreciated in part due to the powerful efficacy of B-cell depletion therapies. An immunotherapy that combines blockade of both the CD40L–CD40 and CD80/86–CD28 pathways, a big part of the conversation, still holds tremendous promise for inducing Ag-specific tolerance.

Ironically, one of the biggest errors that we made was focusing on the G28-5 mAb at the expense of another mAb established in the same fusion, G28-8 (anti-Bgp95). This mAb turned out to bind to a novel member of the Toll-like receptor (TLR) family, CD180/RP105 (40), actually the first mammalian TLR to be cloned and compared to *Drosophila* toll (41). We were not able to isolate the CD180 cDNA by expression cloning since CD180, like its very close relative, TLR4, can only be expressed on the cell surface as a heterodimer (CD180/MD1). Anti-CD180 mAbs coupled to Ags efficiently induce a potent B-cell adjuvant signal and IgG Abs (42), and the combination of viral envelope Ag attached to anti-CD180, can induce protective immunity (unpublished data). Ironically, the at-times forgotten G28-8 mAb may turn out to be as interesting or more interesting than its G28-5 sister.

## Future Clinical Prospects

A number of anti-CD40 Abs and CD40L blocking agents (anti-CD154 or CD40-Ig) are in clinical development for treatment of autoimmune diseases, transplant rejection, and cancers (43). For the treatment of autoimmune diseases and transplant rejection, the goal is to bring forward therapeutics that can block the CD40L–CD40 pathway as part of a “costimulation blockade” strategy, for example, to induce transplantation tolerance (44). Recently, an anti-CD40L engineered so it does not have Fc effector functions like binding to Fc receptors on platelets was shown to block the development in mice of a systemic lupus erythematosus-like disease (45).

CD40 is a particularly attractive oncology target. It is expressed on both lymphoid malignancies and on a range of carcinomas. Thus, anti-CD40 mAbs may help mediate cancer cell killing by effector cells. Furthermore, as seen with the M12 cell line (20, 29), CD40 ligation of some tumor cells can lead to cell death (43, 46). Humanized anti-CD40 mAbs are currently being tested in clinical trials for the treatment of non-Hodgkin’s lymphomas (NHLs) and other cancers (43). The signaling pathway induced by anti-CD40, as we reported with normal B cells (3) is distinct from and synergistic with anti-CD20 mAbs like rituximab, and can promote increased lymphoma cell death (47–49). Thus, CD40-based immunotherapies may find use in conjunction with CD20-based therapies (43) for NHLs as well as with other agents (50).

Furthermore, anti-CD40 mAbs hold great promise for use as part of vaccines against cancers and infectious diseases [e.g., Ref. (51–53)]. In particular, CD40 ligation can promote the activation of cytotoxic CD8 T cells (38), essential for effective therapeutic vaccines. Local administration and slow release of anti-CD40 may mitigate the potential adverse side effects associated with agents that strongly modulate the immune system (51, 52). Thus, it is very likely that one or more new CD40-based therapies will be in use in the coming decade.

## Conflict of Interest Statement

The author declares that the research was conducted in the absence of any commercial or financial relationships that could be construed as a potential conflict of interest.
